# Non-Antibiotic Antimicrobial Catheter Lock Solutions in Patients on Home Parenteral Nutrition

**DOI:** 10.3390/nu10091165

**Published:** 2018-08-25

**Authors:** Jessica Noelting, Brian Jurewitsch, Johane P. Allard

**Affiliations:** 1 Division of Gastroenterology & Gastrointestinal Diseases Research Unit, Queen’s University, Kingston, ON K7L 2V7, Canada; jessica.noelting@kingstonhsc.ca; 2 Specialized Complex Care Program, St. Michael’s Hospital, Faculty of Pharmacy, University of Toronto, Toronto, ON M5B 1W8, Canada; Brian.Jurewitsch@utoronto.ca; 3 Division of Gastroenterology, University of Toronto, Toronto, ON M5G 2C4, Canada

**Keywords:** central venous catheter, parenteral nutrition, catheter-related infections, anti-bacterial agents

## Abstract

Patients on home parenteral nutrition (HPN) are dependent on central venous access for long-term sustenance, and catheter-related bloodstream infections (CRBSIs) are a major cause of morbidity and mortality in this patient population. As such, there is much interest in finding new methods for preventing CRBSIs in patients on HPN. As it is thought that these infections are preceded by microbial colonization of the catheter, one approach is to use antimicrobial catheter lock solutions. Although antibiotic catheter lock solutions have been present for decades, their use has been mostly limited to the treatment of CRBSIs due to concern for promoting microbial resistance. Recently, however, with the advent of non-antibiotic antimicrobial catheter lock solutions, this approach is gaining popularity as a promising method to decrease rates of CRBSI in HPN patients.

## 1. Introduction

Parenteral nutrition is a life-sustaining therapy required in patients who do not have a functioning gastrointestinal tract. This can be either second to surgical removal, or malfunction (such as in malabsorptive conditions and dysmotility). The intravenous provision of nutrition requires central venous access so that a hyperosmolar nutrient solution can be infused. A variety of central venous catheters (CVCs) can be used, including peripherally inserted central catheters (PICCs), tunneled catheters, and implanted ports. The most common complication, and most frequent cause of hospitalization in patients on home parenteral nutrition (HPN) is bloodstream infection [1]. Additionally, repeated infections and replacement of venous access catheters can lead to complications such as the loss of vascular access sites. Therefore, many efforts have been made to reduce the risk of catheter-related bloodstream infections (CRBSIs) including: education in aseptic technique, application of topical antimicrobial agents to the exit site, and reducing the number of manipulations of the catheter and solutions for infusion. Although antimicrobial catheter lock solutions have been used for many years, they were traditionally composed of antibiotics, and due to concern for promoting antibiotic resistant microorganisms, their use was restricted to treatment of infections. Antimicrobial catheter lock solutions are also used in hemodialysis catheters [2] but reviewing this is beyond the scope of this paper.

The aim of this scoping review is to discuss the various non-antibiotic catheter lock solutions, their potential role for CRBSI prophylaxis in HPN patients and to identify the need for further research in this area.

## 2. Non-Antibiotic Antimicrobial Catheter Lock Solutions 

### 2.1. Citrate

Citrate has been successfully used for prevention of thrombosis and CRBSI in dialysis catheters [3]. However, this included citrate in combination with other microbial agents. When evaluating citrate alone, it was not better than heparin for prevention of CRBSI at concentrations up to 4% [3,4]. Only one trial showed that it may have antimicrobial effects, but this was with much higher concentration of 30% [5]. Although citrate alone as a catheter lock solution has not been evaluated in HPN patients, it is frequently combined with other agents. Citrate is thought to have anticoagulant activity and to reduce biofilm, which can aid with the penetration of other antimicrobial agents. The mechanism of action of citrate is related to calcium chelating properties. However, this has also led to concerns regarding the safety of citrate in high concentrations due to the risk of hypocalcemia and risk of bleeding, and it is generally recommended to use a maximum concentration of 4% citrate for catheter lock solutions and to attempt as close a match as possible between the volume of the citrate with that of the catheter. In general, there is some 15% of overspill of citrate into the circulation at time of instillation which may account for why patients may report immediate sensations depending on the concentration [6,7]. It is prudent for these patients to aspirate the lock at the end of the dwell time; however, fibrin sheath formation at the tip of the catheter and obscuring the lumen may function as a one-way valve not permitting aspiration/blood return, leaving slow flushing as the only option in reaccessing the line.

### 2.2. Ethanol

Ethanol, in various concentrations, has been successfully used to prevent and treat CRBSI in HPN patients. There have been many studies showing the value of ethanol lock therapy (ELT) in pediatric patients on HPN, including a meta-analysis combining nine studies of patients with intestinal failure that showed 6.27 fewer events per 1000 catheter days in patients using ELT compared to heparin [8,9]. In a retrospective review of 31 adult HPN patients with tunneled silicon catheters and a history of CRBSI, the rates of CRBSI decreased from 3.53 to 1.65 per 1000 catheter days after initiation of ELT [10]. In that study, patients locked each lumen of their catheter with a 70% ethanol solution after each infusion, and then flushed their catheter with normal saline prior to the next. In another study using this same solution and lock/flush protocol, there was a decreased rate of CRBSI from 4.18 to 0.47 per 1000 catheter days in eight adult HPN patients with single-lumen tunneled catheters made from polyurethane [11]. Although ethanol lock solutions have shown promise in reducing CRBSI, their use has been limited due to higher rates of mechanical complications such as occlusion and disrupted integrity of the catheter, requiring increased catheter replacement [9,12,13]. There is also concern regarding the potential for inebriation with flushing concentrated ethanol into a central vein. General application of ELT for HPN patients in the United States, for example, is also limited by the fact that it does not have the indication for locking catheters from the Food and Drug Administration, making it an off-label use.

### 2.3. Taurolidine

Taurolidine is a derivative of taurine, an amino acid; it degrades into taurine, carbon dioxide and water. It is a bactericidal agent that is effective against gram positive and gram negative organisms, as well as some fungi [4]. There have been many studies showing the effectiveness of taurolidine lock solution for secondary prophylaxis of CRBSI [14,15,16,17,18,19,20,21]. However, as described in meta-analyses, most of these studies are limited by methodology (observational) and small sample size [22]. There are two randomized controlled trials, which will be reviewed here. The first is a randomized, placebo-controlled, double blind study of 41 patients (21 heparin, 20 taurolidine) [19]. In this study, a solution containing taurolidine 1.35%, sodium-citrate 4%, and heparin 100 IU/mL was used, and only patients with a previous CRBSI in the last 4 years and a tunneled catheter were included. None of the patients in the taurolidine arm experienced a CRBSI during the total 9622 catheter days, as compared to seven CRBSIs in the heparin arm during a total of 6956 catheter days. Although there were seven positive blood cultures in the taurolidine group, these were either not treated, or another source of infection was found and treated. Despite the small number of patients in this study, there was a significant reduction in CRBSIs (*p* = 0.0052) in patients receiving taurolidine compared to heparin, without a difference in occurrence of mechanical complications. The second is a multi-center randomized, double-blinded study comparing 2% taurolidine to 0.9% saline in 102 patients from five countries [21]. They included two groups of patients: 71 patients with new central venous catheter and 31 patients which they called ‘high-risk’, defined as having a pre-existing central venous catheter in place for at least 6 months prior to enrollment, on HPN for at least one year and a CRBSI rate of at least 0.82 per 1000 catheter days. They showed that taurolidine significantly decreased rates of CRBSI per thousand catheter days in the new catheter group (0.29 versus 1.49) and in the combined group (0.33 versus 1.44) (*p* = 0.002). Although the rate of CRBSI was also decreased in the high-risk group, this was not statistically significant, possibly due to the small number of patients in that group. However, it is also possible that there was already a biofilm present in those patients’ catheters and that taurolidine was not effective in that setting. Additionally, some of the catheters could have been subjected to salvage protocols with antibiotics having been both intraluminally applied as a lock and intermittently infused to treat with the result of residual high colony counts left in the biofilm, outstripping the effect of the taurolidine over time, although this detail is not disclosed. Forty-four out of 71 new catheter patients had never had a CRBSI prior to enrollment, and, in these patients, none of those randomized to taurolidine experienced a CRBSI, compared to nine of those in the saline arm. It is also notable that, in this study, there was no difference in catheter occlusion rate or patient satisfaction between saline and taurolidine. There is only one study that aimed to assess the use of taurolidine in patients with a low rate of CRBSI (defined as less than one CRBSI per patient per year) [23]. Unfortunately, with only 30 patients followed for one year, divided between three different catheter lock solutions (taurolidine 2%, taurolidine 1.34% with citrate and saline), this study was likely underpowered to demonstrate any difference between the three catheter lock solutions. There are different taurolidine catheter lock solutions used in various studies including either heparin and/or citrate in addition to taurolidine, and with taurolidine concentrations ranging from 1.34% to 2%. However, an in vitro analysis of different taurolidine solutions did successfully inhibit the growth of microbial pathogens [4], and although the microbicidal effect of taurolidine was greater at higher concentration (2% versus 1.34%), the clinical significance of this is not known. This study also demonstrated that the combination of taurolidine with heparin and/or citrate did not affect its antimicrobial effect. As it is not an antibiotic, one would not expect antimicrobial resistance to develop. Accordingly, a study from the Netherlands using 27 study isolates from 9 patients who developed CRBSIs while using taurolidine lock solution did not find any microbial adaptation of microorganisms to taurolidine [24].

### 2.4. Tetrasodium Ethylenediamine Tetraacetic Acid (EDTA)

The tetrasodium EDTA catheter lock solution has been shown to reduce biofilm formation and bacterial colonization [25,26]. Furthermore, EDTA is a known anticoagulant and it is used in blood collection tubes because of this property [27]. The tetrasodium EDTA lock solution has been safely used in hemodialysis catheters [26]; however, there are no published studies of the tetrasodium EDTA catheter lock solution in patients on home PN.

## 3. The Canadian Experience

Currently, patients on home PN in several programs in Canada do not use an antimicrobial catheter lock solution unless they have recurrent (more than one) CRBSIs. In Canada, Taurolidine is currently only approved for investigation use so it can only be obtained after successful application for special access, which requires one application per patient. Furthermore, depending on regions and funding policies, there is variation in the cost of lock solutions which determines the choice of the lock solutions. It is not known which agent between taurolidine, EDTA or citrate is the most cost-effective. One case report provided cost-utility estimates on the use of taurolidine in a Canadian home PN patient suffering from multiple CRBSI episodes which demonstrated a huge spread in cost of using a lock solution vs. the standard of care (heparin) incurring repeated CRBSIs with cost overwhelmingly favoring prevention [28].

## 4. Conclusions

In conclusion, there are several non-antibiotic catheter lock solutions that can be used for antimicrobial prophylaxis, including citrate, ethanol, taurolidine, and tetrasodium EDTA. Although both ethanol and taurolidine have shown reduction in CRBSI rates in high-risk adult HPN patients (Table 1), there is limited evidence regarding the use of a catheter lock solution for primary prophylaxis of CRBSI. The Infectious Disease Society of America does recommend the use of antimicrobial catheter lock solutions for prophylaxis in high-risk patients with long-term catheters. However, one group from the Netherlands reports having placed their entire HPN population on the taurolidine lock solution [24].

Further research is needed to define which HPN patients should be placed on non-antibiotic antimicrobial catheter lock solution for prophylaxis and to determine which solution, or combination of solutions, is optimal. In the future, one might even consider adding glyceryl trinitrate to the catheter lock solution to add an antifungal protection [29].

## Figures and Tables

**Table 1:** Studies of non-antibiotic antimicrobial catheter lock solutions in adult home parenteral nutrition (HPN) patients. CRBSI: catheter-related bloodstream infections.

Authors (Year of Publication)	Study Design	Number of Patients	Intervention	Location	Duration (Days Per Patient)	Outcome	Complications	Notes
Worley, M.V. et al. (2017) [30]	Retrospective cohort	24	70% ethanol, usual care	The United States	362, 235	12.7 versus 2.4 CRBSIs per 1000 catheter days (*p* = 0.004)	None	Ethanol use was instituted in April 2012. Patients compared before and after that date.
Davidson, J.B. et al. (2017) [11]	Retrospective cohort	8	70% ethanol	The United States	Not given	4.18 versus 0.47 CRBSIs per 1000 catheter days	None reported	Data not available individually for ethanol lock therapy.
John, B.K. et al. (2012) [10]	Retrospective cohort	31	70% ethanol, usual care	The United States	878, 232	3.53 versus 1.65 CRBSIs per 1000 catheter days (*p* = 0.011)	None	Each patient served as their own control
Opilla, M.T. et al. (2007) [31]	Retrospective cohort	9	25–70%ethanol, usual care	The United States	Not explicitly stated	8.3 versus 3.7 CRBSIs per 1000 catheter days (*p* = 0.001)	Some patients felt lightheaded and ‘high’ after flush. One patient felt nauseated.	Before-and-after study design.Dwell time 2–4 h.
Wouters, Y. et al. (2018) [21]	Randomized controlled trial	105 randomized, 102 analyzed	2% taurolidine, 0.9% saline	Denmark, Israel, Italy, the Netherlands, the United Kingdom	363, 346	0.33 versus 1.44 CRBSIs per 1000 catheter days (*p* = 0.002)	No difference in rate of catheter occlusion or patient satisfaction	
Tribler, S. et al. (2017) [19]	Randomized controlled trial	41	1.35% taurolidine + 4% citrate + heparin 100 IU/mL, heparin 100 IU/mL	Denmark	481, 331	No CRBSIs in the taurolidine group versus seven CRBSIs in the heparin group (P-log-rank = 0.0034)	No difference in rate of mechanical complications	There were seven positive blood cultures in the taurolidine group but all were classified as contaminants and only one person received antibiotics for a short period
Bisseling, T.M. et al. (2010) [14]	Open-label randomized controlled trial	30	2% taurolidine, heparin 150 IU/mL	The Netherlands	336, 353	0.19 versus 233 CRBSIs per 1000 catheter days (*p* = 0.008)	None	
Klek, S. et al. (2014) [23]	Open-label randomized controlled trial	30	2% taurolidine, 1.35% taurolidine + citrate, 0.9% saline	Poland	365.8, 365, 366	1 CRBSIs in a patient using 1.35% taurolidine + citrate	1 catheter occlusion in a patient using 2% taurolidine	This study was conducted in a group of patients with a low infection rate (0.3–0.4 episodes per patient per year)
Toure, A. et al. (2012) [20]	Retrospective cohort	15	1.35% taurolidine + 4% citrate, 0.9% saline	France	365, 365	6.58 versus 1.09 CRBSIs per 1000 catheter days (*p* < 0.001)	Not available	Eight patients used taurolidine solution only once a week, the others used it after each PN infusion; each patient served as their own control using a before-and-after study design
Saunders et al. (2015) [15]	Retrospective cohort	22	1.35% taurolidine + 4% citrate or 2% taurolidine, 0.9% saline	The United Kingdom	334, 551	5.71 versus 0.99 CRBSIs per 1000 catheter days (*p* < 0.0001)	Not available	Three out of 22 patients were using taurolidine for primary prophylaxis
